# Clinical Reports

**Published:** 1880-03

**Authors:** F. D. Wilson

**Affiliations:** The Dental College


### CLINICAL REPORTS.

Department of Medicine and Surgery, University of Michigan.
Clinic of Prof. Maclean, January 14th, 1880.

*Reported by F. D. Wilson, of the Dental College.*

### EPITHELIOMA.

In this case, the patient has been troubled for the last two years with a large epithelioma, involving the whole of the lower lip, but there was no glandular enlargement. Prof. Maclean removed the growth as follows: after giving an anæsthetic, a V shape incision was made from the angles of the mouth to a point above the symphasis menti, and then two incisions along the lower jaw at right angles with the former two. The V shaped portion of lip was removed; the two points of integument, (one from each side.) were dissected loose, and raised to the level of the former lip, and the remaining point in the median line dissected loose and stretched upward to complete closure of the gap; thus forming a new lower lip. The hare lip needles and silver sutures were used to hold the parts together, and removed a week afterwards. The hemorrhage was very slight and was easily controlled. The operation was a complete success, the parts healed readily and for the most part by first intention. There is no indication of return, now more than a month since the operation.

January 21st, Mr. I, aged 44, Epithelioma, the disease extended from the third molar tooth to the lips, and was reflected on the upper and lower jaws. The submaxillary and sublingual glands were slightly enlarged, the disease had lasted twelve years and he has had no treatment. As is unusual with these cases, the disease originated at the back of the mouth, and grew forwards, and in such cases the prognosis is always more unfavorable. The disease is telling on the general health of the patient. It has grown more rapidly in the last few months, as an operation would involve the removal of part of both jaws and most of the left cheek, and a probability of its return in some other part of the body, the patient was dismissed without an operation.

January 14th, another case was a man aged 56 years, who had been troubled with quite a large cancerous tumor on the right temple, for the last eight years, he had used various domestic remedies, but with no relief, Dr. Maclean recommended that chloride of zinc paste be applied.

